# *In chemico* methodology for engineered nanomaterial categorization according to number, nature and oxidative potential of reactive surface sites†

**DOI:** 10.1039/d3en00810j

**Published:** 2024-07-09

**Authors:** V. Alcolea-Rodriguez, R. Portela, V. Calvino-Casilda, M. A. Bañares

**Affiliations:** a Instituto de Catálisis y Petroleoquímica, ICP-CSIC Marie Curie 2 28049-Madrid Spain valcolear@gmail.com raquel.portela@csic.es miguel.banares@csic.es; b Departamento de Ingeniería Eléctrica, Electrónica, Control, Telemática y Química Aplicada a la Ingeniería, E.T.S. de Ingenieros Industriales, UNED Juan del Rosal 12 28040-Madrid Spain vcalvino@ieec.uned.es

## Abstract

Methanol probe chemisorption quantifies the number of reactive sites at the surface of engineered nanomaterials, enabling normalization per reactive site in reactivity and toxicity tests, rather than per mass or physical surface area. Subsequent temperature-programmed surface reaction (TPSR) of chemisorbed methanol identifies the reactive nature of surface sites (acidic, basic, redox or combination thereof) and their reactivity. Complementary to the methanol assay, a dithiothreitol (DTT) probe oxidation reaction is used to evaluate the oxidation capacity. These acellular approaches to quantify the number, nature, and reactivity of surface sites constitute a new approach methodology (NAM) for site-specific classification of nanomaterials. As a proof of concept, CuO, CeO_2_, ZnO, Fe_3_O_4_, CuFe_2_O_4_, Co_3_O_4_ and two TiO_2_ nanomaterials were probed. A harmonized reactive descriptor for ENMs was obtained: the DTT oxidation rate per reactive surface site, or oxidative turnover frequency (OxTOF). CuO and CuFe_2_O_4_ ENMs exhibit the largest reactive site surface density and possess the highest oxidizing ability in the series, as estimated by the DTT probe reaction, followed by CeO_2_ NM-211 and then titania nanomaterials (DT-51 and NM-101) and Fe_3_O_4_. DTT depletion for ZnO NM-110 was associated with dissolved zinc ions rather than the ZnO particles; however, the basic characteristics of the ZnO NM-110 particles were evidenced by methanol TPSR. These acellular assays allow ranking the eight nanomaterials into three categories with statistically different oxidative potentials: CuO, CuFe_2_O_4_ and Co_3_O_4_ are the most reactive; ceria exhibits a moderate reactivity; and iron oxide and the titanias possess a low oxidative potential.

## Abstract 2

Methanol probe chemisorption quantifies the number of reactive surface sites of engineered nanomaterials, enabling normalization per reactive site in reactivity and toxicity tests, rather than per mass or physical surface area.

## Graphical abstract

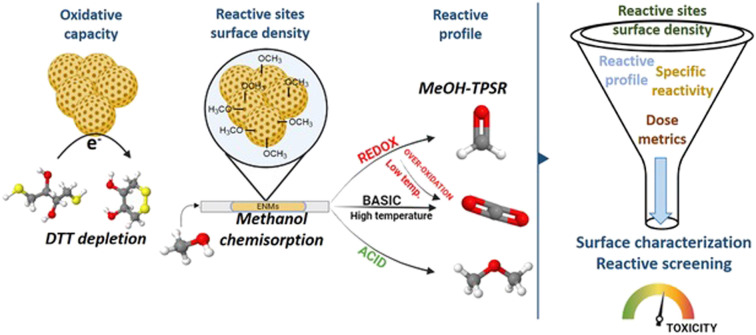

**Environmental significance**

The hazard of nanomaterials is associated with their surface reactivity. We report an *in chemico* approach to probe the number, nature and reactivity of surface sites. This approach demonstrates how many sites we have at the surface of nanomaterials that are relevant for a dose metric based on actual sites rather than on the surface or mass. This would allow for better insight into dose–response investigations. This NAM provides insights into whether sites are oxidative, acidic, basic, or a combination thereof, and additionally aids in ranking NMs by reactivity, which is crucial for understanding their mechanisms of toxicity. In a broader view, it can characterize nanomaterials and how their reactivity evolves as they change, making multicomponent nanomaterials and as they age during operation and in the environment.

## 1 Introduction

### The surface of engineered nanomaterials (ENMs)

**Fig. 1:**
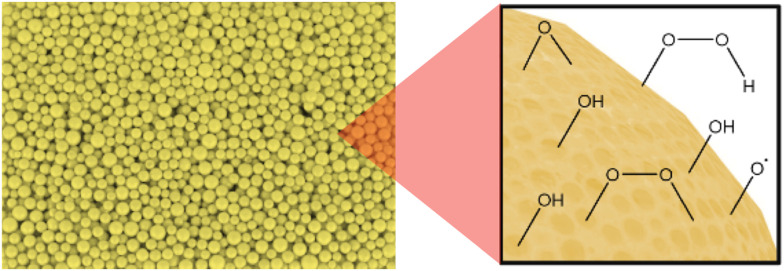
Reactive sites on the ENM surface.

Metal oxides possess a lattice in which the unit cell repeats *ad infinitum*. However, materials are finite and interact with the surrounding environment through their surface, which represents the end of the lattice periodic structure. This surface is characterized by descriptors such as specific surface area (BET area), pore size, or zeta potential (*ζ*).^1^ Surface chemistry defines materials' reactivity (type and strength); in metal oxides, it is often associated with surface oxygen species, such as bridging oxygen, oxide, superoxide, peroxide, or hydroxyl sites (Fig. 1), the properties of which are determined by underlying cations, defects, and the bulk structure. Charge unbalances, such as surface vacancies and defects, are stabilized to maintain the material neutrality, typically by surface interactions, *e.g.*, with environmental water, generating surface hydroxyl groups. The compensation mechanisms for defects may vary depending on the nature of the material. For instance, in ionic materials, defect compensation might involve the the formation of *farb* centers; incovalent ENMs, it could involve covalent; and in transition metal oxides, it may involve transitions between the valance and the conduction band.^2^ Surface relevance is maximized in non-soluble nanomaterials (NMs, with one dimension in the 1–100 nm range),^3^ in which a high surface-to-volume ratio confers them with distinctive properties. For example, in the field of ecotoxicology, 40 mg L^−1^ of nano-sized CuO particles completely inhibit the growth of *S. cerevisiae*, while 4000 mg L^−1^ of CuO bulk material is needed to achieve this;^4^ the number of exposed sites is probably not dramatically different between these two very different amounts of CuO materials.

### Rise of engineered nanomaterials and concerns about their toxicity

Engineered nanomaterial applications have grown significantly, influencing societal challenges and the economy, especially in Asia-Pacific, America, and Europe.^5–7^ Transition metal oxide ENMs, including TiO_2_, CuO, and ZnO, have versatile uses, such as pigments and catalysts.^8,9^ This has prompted numerous characterization, exposure and hazard studies^10–12^ to understand and prevent possible adverse effects or pathologies, for example, those derived from reactive oxygen species (ROS) release,^13,14^ and to adopt a knowledge-based safe-by-design (SbD) approach,^15,16^ which is essential to ensure safe ENM applications as well as faster, more economical and more effective production routes.^17,18^ Integrated information related to toxicity (*in vitro* and *in vivo* testing) and physicochemical properties underpins hazard prediction^16,19,20–23^ with machine learning serving as the primary tool.^24^ Grouping ENMs based on similarities can optimize resource management, aligning with OCDE guidelines for risk assessment and promoting non-animal testing methodologies.^25,26^ New approach methodologies (NAMs) may provide the basis for this objective.^27–29^ The overarching aim is to contribute to the understanding of adverse outcome pathways (AOPs) and, particularly, what reactive properties are associated with the triggering of adverse effects by nanomaterials.^30^

### New approach methodology based on surface site reactivity

Nanomaterial surface reactivity plays a vital role in oxidative-stress-induced adverse effects.^31–34^ Because reactivity is an extrinsic property considered a key parameter to describe the interaction of ENMs with their surroundings,^16,19^ the development of abiotic *in chemico* assays to evaluate surface reactivity and link it with key events in reactive-based nanotoxicity would help to fundamentally understand the modes of action^35–37^ and better group ENMs while minimizing *in vivo* testing.^38^ Surface reactivity characterization complements other physicochemical information relevant to the nanotoxicity field^19^ to investigate toxic ion release, lung fibrosis, inflammasome activation, and interference with embryonic hatching or membrane lysis, among others. In the context of reactive-based toxicity assessment of engineered nanomaterials, it is widely acknowledged that materials with identical chemical compositions can lead to significantly varied biological oxidative damage.^34^ Thus, characterizing the amount, nature and reactivity of surface sites is essential for identifying an additional parameter impacting nanomaterial's effects. The interaction with biological systems depends on the surface properties of the nanomaterials, and the presence of any kind of active site may have effects on their interaction with molecules. We hypothesize that mapping all reactive sites (redox, acidic and basic) may provide a better reactive description of nanomaterials than just oxidative sites and enable a more reliable grouping of nanomaterials based on their surface reactivity. Formally equivalent problems have formally equivalent solutions: as key events in reactive-based toxicity and catalytic reactions occur at the surface; more specifically, at the reactive sites, we propose the use of catalytic methods based on the adsorption and reaction of probe molecules to quantify the surface reactive sites of ENMs and characterize their reactive nature, thus delivering descriptors relevant for ENM classification.^39,40^

Reactive characterization may also provide new dose metrics. In *in vitro* tests with different cell lines, quantitative dose-dependent cellular/biological effects are typically normalized by the mass or physical BET area. These dose metrics may sometimes not be useful to compare exposure because the mass or exposed physical area does not necessarily correlate with the number of reactive sites, which trigger chemical processes, *e.g.*, ROS formation (the generation of ROS by particles is one of the possible molecular initiating events that lead to adverse outcomes, as confirmed, *e.g.*, for PM, CuO, or photo-activated TiO_2_). We do not tackle photocatalytic phenomena that are unlikely to happen inside the body. Research on heterogeneous catalysis has traditionally faced the same challenge when comparing the activities of catalytic materials and has reached a consensus that the most relevant metric is the turnover frequency (TOF). TOF is the number of times that the overall catalytic reaction occurs (*i.e.*, molecules that react) per reactive site and unit time.^39,41–44^ Our research posits that probe molecules allow the quantification of reactive surface sites, their nature and reactivity; TOF calculation can thus be made based on relevant probe reactions (*e.g.*, DTT), offering new metrics for reactivity and toxicological studies.

The most typical probe molecules used to quantify reactive sites in heterogeneous catalysis are carbon monoxide for metal NPs^45^ and methanol for metal oxides, both in the gas/vapor phase. The latter is considered a “smart” probe molecule that can quantify the number of surface sites by chemisorption and report on their reactive profile by temperature-programmed surface reaction (TPSR),^46,47^ a powerful technique to identify and quantify acidic, basic, redox, and bifunctional sites^48,49^ on materials that are not thermally sensitive. In addition, several probe molecules in the liquid phase may specifically assess the oxidative potential (OP),^50–55^ which is particularly relevant to human health due to its involvement in cellular damage caused by oxidative stress.^10,56–60^ Among them, dithiothreitol (DTT) depletion is suggested here as an acellular, liquid-phase, low-temperature probe reaction to assess the nanomaterial OP because this molecule has been previously used to quantify the oxidative capacity of particulate matter.^61–66^ We therefore introduce a NAM using gas-phase methanol chemisorption and subsequent TPSR as well as liquid-phase DTT consumption in PBS-water solutions with nanomaterials. By normalizing the DTT oxidation rate *via* methanol chemisorption, we derive the oxidative turnover frequency (OxTOF) to measure surface site reactivity. We suggest dose normalization of the amount and reactivity of the reactive sites. As a proof of concept, seven metal oxide nanomaterials (CeO_2_, ZnO, CuO, Fe_3_O_4_, Co_3_O_4_, and two TiO_2_ variants), one bimetallic nanooxide (CuFe_2_O_4_) and an oxide with larger particles (Co_3_O_4_) are analyzed to investigate the usefulness of this NAM to 1), categorize the reactivity of eight benchmark engineered nanomaterials; 2) assess the differences in reactivity between ENMs with the same composition (TiO_2_ NM-101 *vs.* TiO_2_ DT-51); 3), assess the effect of bimetallic compositions on the surface reactivity of metal nano-oxides (monometallic *vs.* bimetallic); 4), calculate reactive rankings according to three dose metrics based on mass, surface area and surface sites; and 5), assess the size-dependent reactivity of a material by comparing Co_3_O_4_ nanoparticles to its larger counterpart.

## 2 Experimental

### 2.1 Nanomaterials

All nanomaterials were used as supplied. Two anatase TiO_2_ powders were compared: DT51 (CristalACTiV™) and NM-101 (labeled JRCNM01001a by the supplier, the Joint Research Centre, JRC). In addition, two more JRC samples: CeO_2_ NM-211 (JRCNM02101a) and ZnO NM-110 (JRCNM62101a), as well as four commercial samples from Sigma-Aldrich (CuO (ref. number: 544868, CuO-SA), CuFe_2_O_4_ (ref. number: 641723, CuFe_2_O_4_-SA), Fe_3_O_4_ (ref. number: 637106, Fe_3_O_4_-SA), and Co_3_O_4_ (ref. number: 637025, Co_3_O_4_-SA)) were evaluated. Table S1† summarizes the data and information on these proof-of-concept samples. The size dependence of reactivity was evaluated using Co_3_O_4_ microparticles (ref. number: 221643, Sigma-Aldrich <10 μm).

### 2.2 Specific surface area

The specific surface area was calculated by applying the BET method with data obtained in Micromeritics ASAP 2020 adsorption isotherm equipment. All ENMs were pretreated by degassing under vacuum for 16 h at 120 °C before nitrogen adsorption at liquid nitrogen temperature.

### 2.3 Methanol chemisorption and subsequent temperature-programmed surface reaction (TPSR)

Methanol chemisorption/TPSR procedure (see a detailed description in the ESI,† Fig. S1A and S2) is made on a clean dehydrated sample. 100–250 mg of nanomaterial (aggregated samples with aggregates ranging from 25 to 100 μm) were diluted with 500 mg of inert SiC (black 180, Navarro SiC S.A.) under isothermal conditions and placed in a fixed-bed reactor (0.4 cm internal diameter). The sample is first pretreated by heating from room temperature to 450 °C at 10 °C min^−1^ in a 150 mL min^−1^ synthetic air flow and kept at this temperature for 35 min to ensure the removal of moisture and burn away impurities from its surface. After pretreatment, the sample is cooled to 100 °C (or 50 °C for highly reactive ENMs) in synthetic air. After such treatment, the surface remains hydroxylated, but not hydrated; next, the flow feed is switched to argon (100 mL min^−1^) purge. The chemisorption temperature was optimized to prevent the formation of multilayers in the case of highly reactive materials, looking for a balance between methanol condensation at lower temperatures and methanol reaction at higher temperatures, either of which would lead to an overestimation of the surface sites.^67,68^ After purging, at 100 °C (or 50 °C for highly reactive ENMs), 100 mL min^−1^ of 2000 ppm methanol in argon with 5% helium is fed until saturation, as determined by online mass spectrometry residual gas analysis (*cf.* ESI†). The 5% helium in the argon stream is used as an internal reference for online mass spectrometry. The methanol vapor chemisorbs titrating surface hydroxyl groups; this process converts the CH_3_OH molecule into a chemisorbed CH_3_O-moiety. The missing hydrogen atom reacts with the surface hydroxyl, thus releasing an H_2_O molecule per CH_3_OH molecule that chemisorbs. We monitor the effluent gases by applying a quadrupole residual gas analyzer Pfeiffer OmniStar mass spectrometer. The *m*/*z* values followed were CH_3_OH (methanol) = 31, HCHO (formaldehyde) = 30, CH_3_OCH_3_ (dimethyl ether, DME) = 45, CH_3_OOCH (methylformate) = 60, (CH_3_O)_2_CH_2_ (dimethoxy methane) = 75, H_2_O (water) = 18, and CO_2_ (carbon dioxide) = 44. Blank tests were performed with 500 mg of inert SiC (Fig. S3†). Details on the procedure, the calculation of the reactive surface sites and the surface reactions in methanol-TPSR (eqn (S1)–(S5)) are available in the ESI† material. This methodology is limited to thermally stable samples, such as metal oxides.

### 2.4 DTT consumption assay

DDT catalytic oxidation was performed using a batch reactor for 1 h. First, a 200 μg mL^−1^ suspension of ENM in 1 mM phosphate buffer is obtained by sonication, following NanoGenoTox SOP (16 min at 400 W and 10% amplitude).^69^ 3 mL of the ENM suspension is incubated for 1 h at 37 °C and 500 rpm with 3 mL of 100 μM DTT, obtaining a 6 mL reaction mixture with 100 μg mL^−1^ of ENM and 50 μM DTT. Then, the nanoparticles are removed by filtration, and the filtrate, with the unreacted DTT and the reaction products, is mixed with an equal volume of 1 mM Ellman's reagent (5,5′-dithiobis-(2-nitrobenzoic acid), DTNB) to quantify the non-oxidized DTT (Fig. S1B†). Ellman's reagent reacts with the thiol groups (–SH) of the free DTT molecules, forming 5-mercapto-2-nitrobenzoic acid, a colorful complex that is measured at 412 nm by UV-vis spectrophotometry (Shimadzu, UV-2100). In parallel, as a negative control, DTT in phosphate buffer without ENM is incubated under the same conditions and mixed with the Ellman's reagent to evaluate the DTT consumed by direct reaction without catalyst. Hydrogen peroxide 30% (w/w) in H_2_O is used as a positive control because it provides DTT conversion similar to that of 1,4-naphthoquinone,^62^ which is safer and does not require filtration. All reactions were performed in triplicate. Linearity in the measurements at 412 nm of the DTT-DTNB nm complex was calibrated (Fig. S4†). The DTT oxidative potential is expressed as DTT conversion (eqn (1)), as a normalized index of oxidant generation using hydrogen peroxide as a positive control (eqn (2)), or as the DTT reaction rate, normalized *vs.* mass (eqn (3)), *vs.* the ENM surface area (eqn (4)), or *vs.* the number of reactive sites (eqn (5)), *i.e.*, OxTOF.1
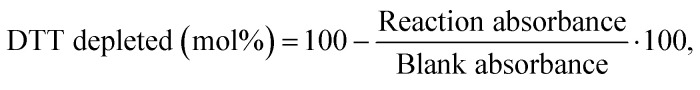
2
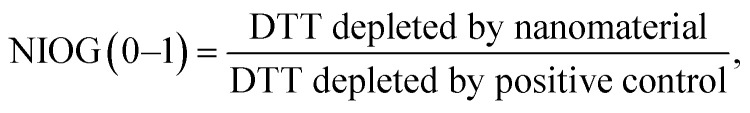
3
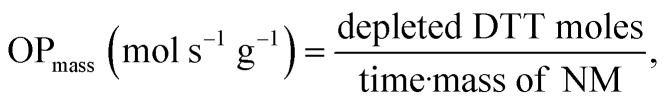
4
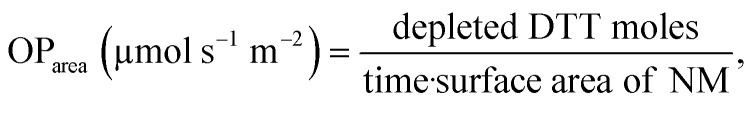
5
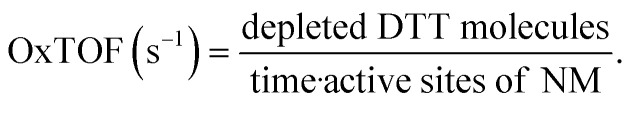


### 2.5 Statistical analysis

DTT OP_mas_, OP_area_ and OxTOF are expressed as average ± sd (standard deviation). The statistical analysis was performed with SPSS 20 (IBM, Armonk, USA) using logarithmic values to obtain a better normal distribution. One-way ANOVA (analysis of variance) was performed to determine statistically significant differences. Subsequently, a Tukey test was performed to assess pairwise differences with a significance level of *p* < 0.05 and classify ENMs according to the oxidative potential.

## 3 Results

### 3.1 Surface area and reactive sites

**Fig. 2:**
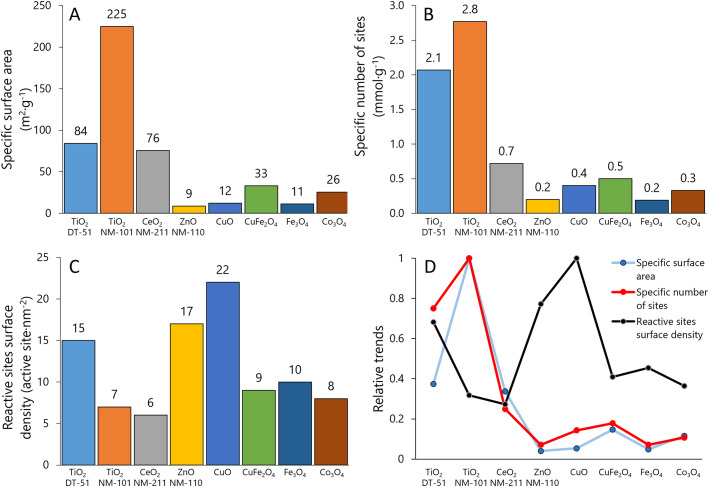
Surface analysis: A) specific surface area obtained by N_2_ adsorption isotherm, B) specific number of reactive sites obtained by methanol chemisorption, C) reactive site surface density obtained by combination of A and B, and D) comparison of the three surface descriptors (values normalized to the maximum).

In the series, TiO_2_ NM-101 exhibits the largest BET area, 225 m^2^ g^−1^; the rest of the ENMs have significantly lower BET values: TiO_2_-DT51, 84 m^2^ g^−1^; CeO_2_ NM-211, 76 m^2^ g^−1^; CuO-SA, 12 m^2^ g^−1^; ZnO NM-110, 9 m^2^ g^−1^; CuFe_2_O_4_-SA, 33 m^2^ g^−1^; Fe_3_O_4_-SA, 11 m^2^ g^−1^; and Co_3_O_4_-SA, 26 m^2^ g^−1^ (Fig. 2A). These data are consistent with the values reported in the literature and the supplier's technical sheets.^70–74^Fig. 2B illustrates the specific number of sites (mmol g^−1^). Although the order of materials remains similar to BET area, the relative values change significantly between Fig. 2A and B. Thus, the reactive site surface density (site per nm^2^) may follow a different trend. Fig. 2C shows this surface descriptor calculated from the data shown in Fig. 2A and B. Interestingly, the ENMs with smaller surface areas have higher reactive site surface densities: 16.6 and 21.8 sites per nm^2^ for ZnO NM-110 and CuO-SA, respectively (values obtained at 50 °C). These data show that due to differences in site types and/or distribution, the numbers of surface area and reactive sites do not linearly correlate for these samples, as might be erroneously assumed. It is remarkable that TiO_2_-DT51, with a specific surface area 2.7 times lower than that of TiO_2_ NM-101, doubles its reactive site surface density (14 *vs.* 7 sites per nm^2^). Therefore, BET (physical) may not be the most relevant descriptor of ENM surface chemistry. Fig. 2D summarizes the trends shown in Fig. 2A–C after data normalization to the most described nanomaterial in the literature: TiO_2_ NM-101.

### 3.2 Reactive profile

**Fig. 3:**
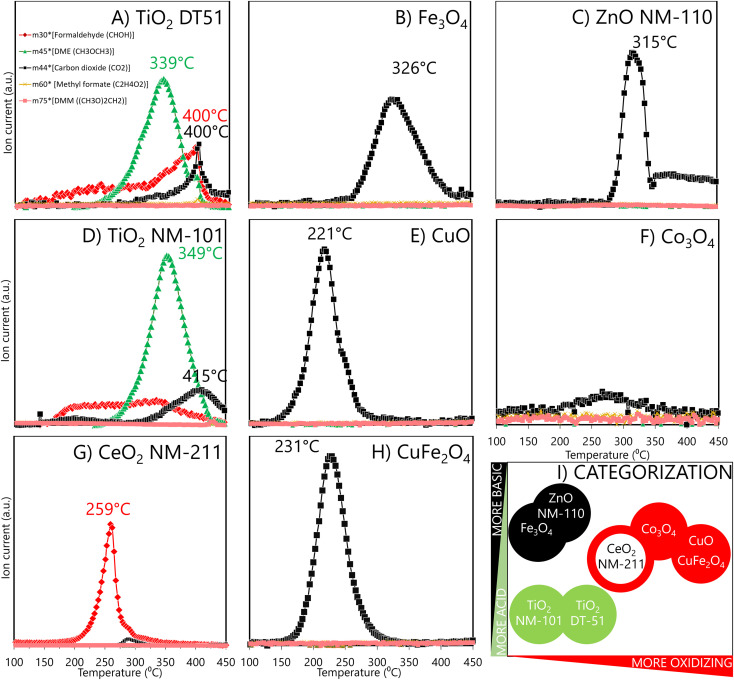
Temperature-programmed surface reaction products of pre-adsorbed methanol analysed by mass spectroscopy for two different anatase TiO_2_: DT51 (A) and NM-101 (D), for Fe_3_O_4_-SA (B), for ZnO NM-110 (C), for CuO-SA (E), for Co_3_O_4_-SA (F) for CeO_2_ NM-211 (G), and for CuFe_2_O_4_-SA (H). Formaldehyde signal (red) is obtained for redox sites, a dimethyl ether signal (green) for acid sites, and carbon dioxide (black) for basic or high reactive redox sites. ENM classification by MeOH-TPSR results is shown in I) with the same colour code. For a given colour, filled circles are more reactive than empty circles.

The methanol TPSR profiles in Fig. 3 provide information on the reactive sites and their reactivity. The typical TPSR products are DME, HCHO and CO_2_.

TiO_2_ ENMs (DT51 and NM-101) mainly form **dimethyl ether** (Fig. 3A and D), the characteristic product of acidic reactivity. The maximum production of dimethyl ether occurs at 349 °C for NM-101 and at 339 °C for DT51, indicating a weaker acidity of the sites of the latter, which are also fewer, as indicated by the smaller area under the curve. Redox (HCHO) and basic (CO_2_) reaction products also form on both titania samples. The redox site is active in a broad temperature range, which indicates a broad distribution of oxidation reactivities, and that the oxidation capacity is moderate because rather high temperatures are required to express it.

Oxidative sites produce **HCHO**. Thus, CeO_2_ exhibits redox sites where methanol is oxidized to formaldehyde, with a maximum near 259 °C (Fig. 3G); ceria oxidative sites exhibit a narrower peak, indicating that most oxidizing sites have similar reactivity. This is unlike the broad distribution of oxidative site types on the titanias in the 150 to 400 °C range. Moreover, ceria has a higher oxidation capacity because its maximum is at a lower temperature than the average of HCHO formation on the titania samples.

ZnO NM-110, CuFe_2_O_4_-SA, Fe_3_O_4_-SA, Co_3_O_4_-SA and CuO-SA (Fig. 3C–H) produce mainly **CO**_**2**_, but the temperatures at which CO_2_ reaches a maximum differ significantly; for some, it is near 220–250 °C, and for others, it is above 300 °C. The significantly higher maximum temperature for CO_2_ production indicates basic materials, where the methoxy species adsorb strongly and can only desorb at very high temperatures before being combusted (ZnO NM-110 and Fe_3_O_4_-SA). Instead, the easier formation of CO_2_ (some 100 °C lower temperatures) indicates a high oxidation capacity, leading to total oxidation of CO_2_ rather than to partial oxidation formaldehyde (CuO-SA, CuFe_2_O_4_-SA and Co_3_O_4_-SA).^49^ Evaluated as a reference, micrometric Co_3_O_4_ (Fig. S5†) showed a similar reactive profile to Co_3_O_4_ nanoparticles. Thus, the nature of the surface sites in nano and micro CuO remains essentially alike, with the critical difference that a minimum part of the reactive sites is exposed in the larger CuO particles; hence, the risk of exposure to larger CuO particles is minimized. In summary, methanol TPSR reactive profiles may classify materials based on a linear combination of their acidic/basic reactive profile *vs.* their oxidation profile (Fig. 3I). In this categorization, the *X*-axis qualitatively indicates how oxidizing the material is, while the *Y*-axis moves from an acidic to a basic character. Thus, TiO_2_ DT51 and NM-101, ZnO NM-110, and Fe_2_O_3_-SA have moderate oxidation capacities, while Fe_2_O_3_-SA and ZnO NM-110 are more basic, and both TiO_2_ variants are more acidic. However, CeO_2_ NM-211 and Co_3_O_4_-SA exhibit increased oxidation capacity, and the highest is for CuO-SA and CuFe_2_O_4_-SA.

### 3.3 Oxidative potential

**Fig. 4:**
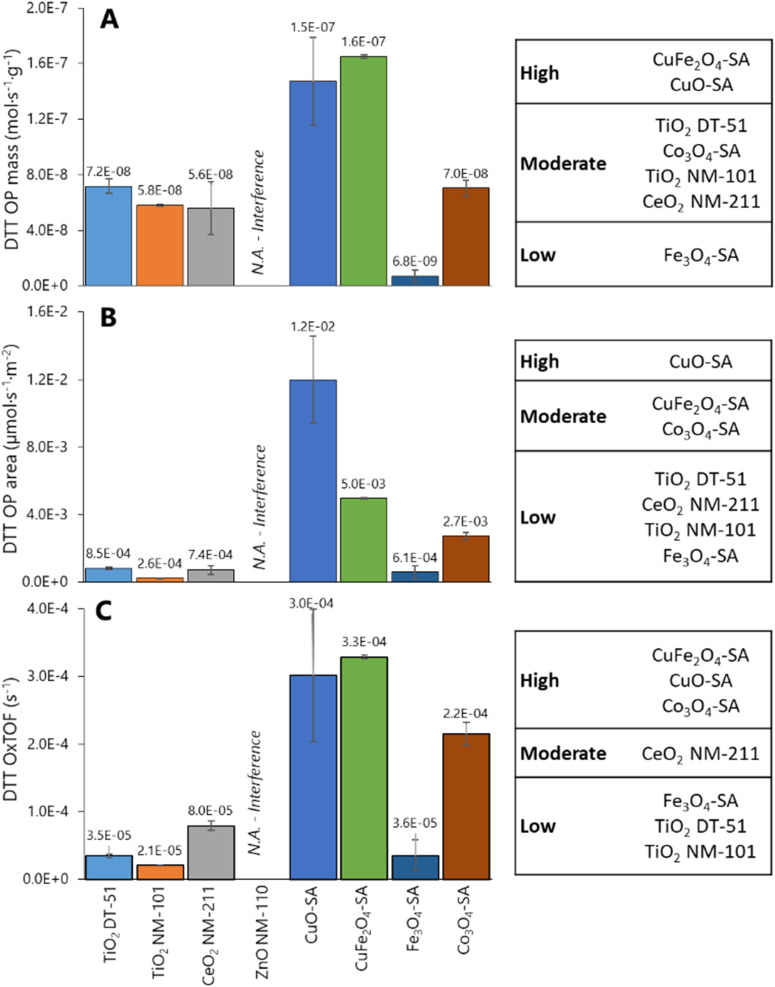
Oxidative potential evaluated by DTT assay and expressed as reaction rate per mass (A), reaction rate per surface (B), and oxidative turnover frequency (reaction rate per reactive site) (C). Left: Averaged OP values (*n* = 3) with error bars indicating the standard deviation. Right: Statistical analysis for classification of the ENMs by OP based on Tukey's test comparison.

Fig. 4 illustrates DDT catalytic oxidation results for 1 h reaction, normalized *vs.* different descriptors; the corresponding classification of the ENMs based on Tukey's test using the logarithm of OP_mass_, OP_area_ and OxTOF is provided on the right side of the plots. ZnO NM-110 is not included in the analysis because it dissolves in the reaction media and Zn cations are complexed by DTT;^66^ therefore, no free and uncomplexed DTT is available for interaction with the ZnO NM-110 surface, and thus the results are close to the negative control.^66^ The relative oxidative potential of the other ENMs significantly depends on the descriptor. The positive control normalization has little impact on the relative conversion trend (Fig. S6†), which is similar to that of the specific reaction rate shown in Fig. 4A: CuO-SA ≈ CuFe_2_O_4_-SA ⋙ Co_3_O_4_-SA ≈ TiO_2_ NM-DT-51 ≈ TiO_2_ NM-101 ≈ CeO_2_ NM-211 > Fe_3_O_4_-SA. According to Tukey's test, only CuO-SA and CuFe_2_O_4_-SA are classified as significantly highly reactive ENMs. The differences between the reactivities of these ENMs are clearly amplified when the oxidation rate is normalized to the ENM surface area (Fig. 4B), which underlines that the CuO-SA surface, being small (Fig. 2A), is significantly more reactive than other ENM surfaces in the series. Actually, Tukey's test reveals three reactivity groups of ENMs according to OP_area_ descriptor: CuO-SA > Co_3_O_4_-SA ≈ CuFe_2_O_4_-SA > TiO_2_ DT-51 ≈ CeO_2_ NM-211 ≈ TiO_2_ NM-101 ≈ Fe_3_O_4_-SA. Mass or BET normalizations cannot tell how reactive each site is, so this trend assumes that all physical areas are equally populated by equally reactive sites, which is not the case. Normalization per reactive site (Fig. 4C) delivers the OxTOF, which shows that the CuO-SA and CuFe_2_O_4_-SA sites are the most oxidizing sites, followed by Co_3_O_4_-SA, and *ca.* fourfold more reactive than the ceria sites. The remaining group of materials exhibits significantly lower oxidation activity according to Tukey's test: CeO_2_ NM-211 > TiO_2_ DT-51 ≈ TiO_2_ NM-101 ≈ Fe_3_O_4_-SA. A larger number of sites can make up for an individual site's lower reactivity; therefore, both pieces of reactivity information, global (per material dose) and individual (per site), are important to understand and classify ENMs. Among these descriptors, only OxTOF can identify with statistical significance that ceria has more reactive redox sites than titania ENMs. From a chemical perspective, turnover frequency values may allow for a better quantitative comparison of oxidative potential and provide more accurate insight on reactivity at a molecular scale.

Size dependence of reactivity was evidenced *via* comparison of Co_3_O_4_ nanoparticles *vs.* microparticles, which exhibited a DTT depletion of 6.7 ± 4.2% of DTT depletion, equivalent to a NIOG of 0.08 ± 0.04, that is, around 7 times lower oxidative capacity than its nanoparticle counterpart. This is essentially due to the significantly smaller fraction of reactive sites exposed.

### 3.4 Dose metrics applied to bibliographic toxicological data

Bibliographic toxicity information for TiO_2_ NM-101, CeO_2_ NM-211, ZnO NM-110, CuO-SA, Fe_3_O_4_, CuFe_2_O_4_ and Co_3_O_4_ was extracted from eNanoMapper^75,76^ and the literature to investigate possible correlations of the surface reactivity with *in vitro* toxicity descriptors (Table S2†).

#### CuO-SA

CuO-SA is highly toxic to pulmonary cells, causing cell death and impairing cell functions after 24 hour exposure.^77^ The mechanism involves ion release, autophagy activation, and increased lipid peroxidation.^78–80^ Animal models support lung inflammatory effects but do not show teratogenic potential.^81,82^ In terms of dose metrics, this ENM, the most oxidant in the series, exhibits significant effects on A549 cell viability at 5, 10 and 17.75 μg mL^−1^ gravimetric doses, equivalent to 2, 4 and 7.1 μmol L^−1^ site doses in different studies.^78^

#### CuFe_2_O_4_-SA

CuFe_2_O_4_-SA cytotoxic effects on human lung (A549) and liver (HepG2) cells were analyzed, illustrating dose-dependent toxicity in a concentration range of 10–100 μg ml^−1^ (*i.e.*, 5–50 μmol site per L). Key observations include mitochondrial membrane potential (MMP) depletion, upregulation of the caspase-3 gene, and increased caspase-3 enzyme activity, suggesting apoptotic cell death as a consequence of exposure to these ENMs. Furthermore, an imbalance in cellular redox status was evident through the induction of ROS and depletion of glutathione (GSH), indicating oxidative stress as a potential underlying mechanism of cytotoxicity.^83^

#### ZnO NM-110

ZnO NM-110, extensively studied *in vitro*, exhibits adverse effects in multiple cell lines, with immune system alterations observed in Raw 264.7 and MH-S macrophages (EC50: 10–25 μg mL^−1^, *i.e.*, 3–7.5 μmol L^−1^ sites doses); pulmonary cell lines display cytotoxic and genotoxic effects (LC50: 76 μg mL^−1^, that is, 22.8 μmol sites per L),^84,85^ and respiratory and male reproductive cell lines are affected (EC50 < 20 μg mL^−1^, significantly less than 6 μmol sites per L).^86^ Hepatic damage in C3A was evidenced through the WST-1 test.^87^ Proteomic analysis in NRK-52E reveals pronounced effects, particularly in actin carbonylation; this ENM is classified as highly cytotoxic and a protein carbonylation agent.^88^ Caco-2 cell lines exhibit cytotoxicity due to dissolved Zn^2+^, and HUVEC cell lines show reduced mitochondrial viability attributed to intracellular Zn ions and ROS. In contrast, TiO_2_ NM-101 showed no cytotoxicity or inflammatory markers.^89,90^

#### TiO_2_ NM-101

TiO_2_ NM-101 exhibited no significant cytotoxicity in the A549, HepG2, HK-2, and C3A cell lines. However, the C3A cells showed IL-8 release, indicating inflammation.^87,91^ BEAS-2B cell viability was unaffected by TiO_2_ at concentrations in the range of 1–100 μg mL^−1^, *i.e.*, 2.8–280 μmol sites per L, but DNA damage and IL-6 release were observed at 10 (28 μmol sites per L) and 100 μg mL^−1^, respectively. RAW 264.7 macrophages exposed to TiO_2_ NM-101 released IL-6 and TNF-α at higher concentrations (100 μg mL^−1^).^92^

#### CeO_2_ NM-211

CeO_2_ NM-211 the toxic mechanism of **CeO**_**2**_**NM-211** remains unclear, but protein aggregation and fibrillation are proposed hypotheses.^93,94^ CeO_2_ NM-211 induced moderate pro-inflammatory cytokine release in rat precision-cut lung slices (PCLuS) at 100 μg mL^−1^, representing a site concentration of 70 μmol L^−1^. This is in line with other *in vivo* studies, where inflammatory markers increased in the bronchoalveolar lavage fluid (BALF) after 14 days of exposure.^95,96^*In vitro*, A549 cells exposed to similar cerium oxide nanoparticles at concentrations up to 100 μg mL^−1^ showed no cytotoxicity.^97^ In contrast, NR8383 alveolar macrophages exhibited cytotoxicity at 90 μg mL^−1^ (63 μmol site per L) along with signs of inflammation, including TNF-α release, after CeO_2_ NM-211 exposure.^98^ These results are analyzed in subsection 4.2 with respect to our *in chemico* method, as gravimetric, surface, and reactive site-based concentrations offer insights into the diverse doses of ENMs in toxicology.

#### Fe_3_O_4_-SA

Fe_3_O_4_-SA showed no adverse effects on A549 cell viability for 24–72 h at concentrations of up to 100 μg mL^−1^ (equivalent to 20 μmol site per L), even after being internalized within the cells following 12 h exposure. Furthermore, an increase in lysosomal activity was not detected after 6 h. However, a concentration-dependent decrease in mitochondrial membrane potential at 100 μg mL^−1^ was statistically significant. There was no induction of pro-inflammatory cytokine secretion, including IL-1β, IL-6, IL-8, and TNF-α. This evidence suggests that iron oxide exhibits low cytotoxicity.^99^

#### Co_3_O_4_-SA

Co_3_O_4_-SA cyto-genotoxic and inflammatory responses of **Co**_**3**_**O**_**4**_**-SA** in human alveolar (A549) and bronchial (BEAS-2B) cell lines were evaluated at concentrations ranging from 1–40 μg ml^−1^, which is equivalent to 0.3–12 μmol site per L. Notably, A549 cells exhibited no cytotoxicity, while BEAS-2B cells showed reduced viability at 40 μg ml^−1^ and early membrane damage at 1, 5, and 40 μg ml^−1^. Significant direct and oxidative DNA damage was observed in A549 cells at 20 and 40 μg ml^−1^, with no impact on cytokine release. Conversely, the BEAS-2B cells exhibited significant direct DNA damage at 40 μg ml^−1^ and notable oxidative DNA damage at lower concentrations, coupled with increased TNF-α and IL-8 release at specific concentrations and exposure times. These results underline the differential cellular responses to cobalt oxide nanoparticles, highlighting the enhanced sensitivity of BEAS-2B cells to cytotoxic, genotoxic, and pro-inflammatory effects.^100^ The genotoxic effects of cobalt oxide in Chinese hamster lung fibroblast (V79) cells, primarily mediated by reactive oxygen species, were used to compare with their bulk counterparts: Co_3_O_4_-SA nanoparticles exhibit pronounced genotoxic effects compared to bulk Co_3_O_4_ macroparticles due to significant cytotoxicity and DNA damage attributed to enhanced ROS generation. The mitigation of genotoxic effects with *N*-acetylcysteine, a ROS scavenger, further confirms the central role of ROS in nanoparticle-induced toxicity. Nano-sized particles facilitate closer cellular interactions, leading to significant cytotoxicity and DNA damage from ROS, unlike the minimal interaction and impact observed with bulk materials.^101^

## 4 Discussion

### 4.1 ENM surface sites and reactivity

The exponential increase in surface-to-volume ratio as the particle size decreases to a few nanometers is crucial. Additionally, quantum confinement and discrete energy levels alter electronic states and surface reactivity. We focus on the phenomenological consequences of this, rather than on its origins. A comprehensive categorization of ENM requires an understanding of their reactivity characteristics: the number of reactive surface sites, their reactive nature and relative reactivity. Nanomaterials can oxidize molecules directly or generate ROS through interactions with biological systems, which may alter their properties. These interactions depend on the surface properties of the nanomaterials, including the reactive sites beyond the physical surface area. Although oxidative potential is often highlighted, acidic and basic sites also significantly impact molecular interactions. Our method maps all reactive sites to provide a comprehensive description of nanomaterials and to enable more reliable grouping based on their chemical surfaces. According to our data, reactivity-triggered nanotoxicity depends not only on the number and reactivity of redox sites but also on the presence of basic and acidic surface sites. Their interplay determines how ENMs interact with the environment and physiology. Parameters, such as site-specific numbers and TOF (*e.g.*, DTT OxTOF), are essential in elucidating the reactive potential of ENMs and linking them to potential adverse effects. Additionally, TPSR profiles provide insights into the relative presence and reactivity of these sites, influencing toxicity profiles. The OxTOF tendency in Fig. 4C shows CuO-SA ≈ CuFe_2_O_4_-SA > Co_3_O_4_-SA ≫ Ce_2_O_3_ NM-211, in line with the oxidation capacity assessed by methanol-TPSR. Following this correlation, the titanias, Fe_3_O_4_-SA and ZnO NM-110, exhibit very little methanol oxidation. Therefore, the DTT probe reaction is oxidative dehydrogenation, forming a disulfide group that appears to run mechanistically parallel to the oxidative dehydrogenation of methanol to formaldehyde in TPSR experiments. The use of chemisorption avoids interference from ion release, buffer reactivity (as observed with some probe reactions in phosphate medium),^102^ or agglomeration that occurs in liquid-phase assays.

Reactive site surface density quantified by methanol chemisorption in our series is consistent with values reported in the literature for oxide ENMs, ranging from 0.4 to 22 sites per nm^2^, but typically up to *ca.* 7 sites per nm^2^, corresponding to a monolayer.^39^ The high number of reactive sites on CuO-SA (22) and ZnO NM-110 (17) surfaces must be related to a highly reactive interaction with chemisorbed methoxy groups. Multilayer formation is likely on ZnO NM-110 basic sites, as suggested for La_2_O_3_, MgO or Cr_2_O_3_,^39^ whereas CuO-SA is a highly oxidizing material that transforms surface methoxy groups into formate groups.^103–105^ This is consistent with the extensive CO_2_ desorption profile during MeOH-TPSR. Hence, the chemisorption temperature was set to 50 °C in these materials. CO_2_ formation at low temperatures on highly reactive CuO-SA is characteristic of formate decomposition, while the formation of CO_2_ at high temperatures on alkaline ZnO NM-110 is associated with the decomposition of carbonates.^104^ The determination of reactive sites provides complementary insight to ROS determination probes, whose sensitivity depends on different features. For instance, the basic character of ZnO NM-110 and its high reactive site surface density correlates with the ferric reduction ability of serum (FRAS) assay, an indirect measurement of ROS by total antioxidant depletion, and protein carbonylation assay.^106^ In another study, electron spin resonance (ESR) spectroscopy with 3-carboxy-2,2,5,5-tetramethylpyrrolidine 1-oxyl (CPH) and 5,5-dimethyl-1-pyrroline *N*-oxide (DMPO) probe molecules determined the oxidative potential of ENMs by determining the ROS production.^107^ Little ROS were produced by CeO_2_ NM-211 and ZnO NM-110 when compared with CuO,^106^ which agreed with our reactive ranking based on OxTOF data, and the higher number of reactive surface sites of the latter. The CPH spin probe (more sensitive to singlet oxygen, superoxide radicals, and peroxynitrites) revealed higher ROS production by ceria than by zinc oxide, whereas the DMPO spin trap (more sensitive to hydroxyls and superoxide radicals) showed the opposite trend. Raman spectroscopy, which is highly sensitive to peroxide-related species, can be used to further analyze this. *In situ*^108,109^ and *operando*^110^ Raman spectra show that superoxide and peroxide species are generated at the surface of different ceria materials by interaction with molecular oxygen, but there are no reports of superoxide species formed at the surface of ZnO and TiO_2_. Thus, reactive superoxide species only account for DMPO and CPH by CeO_2_ NM-211 but not by ZnO NM-110 or titania; therefore, ZnO NM-110 must generate more hydroxyls than CeO_2_ NM-211 to account for the DMPO probe results.

Titania, the least-reactive material in our series, highlights the complexity of categorizing nanomaterials. Even with the same composition (TiO_2_) and crystalline phase (anatase), titania samples differ significantly in BET surface area, reactive site density, and strength. TiO_2_ NM-101, as measured by terephthalic acid assay, generates ROS upon photoirradiation but not in the dark.^111^ Conversely, ROS generation detected *via* the DMPO trap was significantly higher than the control not only upon irradiation but also in the dark, although to a lesser extent.^111^ Several studies on titania reactivity and photoreactivity highlight the impact of species in biological systems (*e.g.*, carboxylic acids, amines) that strongly adsorb onto titania surfaces, affecting reactivity.^102,112^ This underscores the importance of characterizing all surface reactive sites: acidic, basic, and redox. The band gap of metal nano-oxides, crucial for correlating with oxidative stress and pulmonary inflammation from photocatalytic ENMs, strongly depends on particle size, nuclearity, and the nature of nearby elements, serving as an indicator of increasing quantum effects.^113–115^

The strong influence of the titania structural variety on its surface reactivity is described from the perspective of nanoinformatics,^116,117^ which uses computational approaches to understand the surface structure and reactivity of ENMs, using this data in a FAIR (findability, accessibility, interoperability and reusability) implementation for the nanosafety community.^118,119^

### 4.2 Oxidative surface sites and *in vitro* cell viability

As *in vitro* assays monitor different effects (cell viability, protein release, inflammation, *etc.*) in specific cell lines (A549, dTHP-1, *etc.*) and do not provide information about biodistribution, biopersistency or biotransformation^23^ of nanomaterials, they are limited in predicting the overall toxicological profile, comparisons are not straightforward, and correlations with physicochemical properties of ENMs can only be done as a first approximation.^77,120^ The *in vitro* toxic effects of ENMs, which over-oxidize methanol to CO_2_ in TPSR and show redox surface reactivity (Co_3_O_4_-SA, CuFe_2_O_4_-SA and CuO-SA), significantly affected different cell lines,^83,101,120^ underscoring the implication of reactive surface sites in nanotoxicity field. When CuO-SA and ZnO NM-110 are compared, the higher reactive site surface density and the lower temperature of maximum methanol conversion to CO_2_ of the former indicate a higher reactivity of CuO-SA, which correlates with the higher toxicity reported by cell viability assays with the A549 line: EC_50_ for 24 h exposure is 17.75 for CuO-SA and 76 μg mL^−1^ for ZnO NM-110.^84,120^ Site-based dose metrics underlines the higher *in vitro* toxicity of CuO-SA sites. DTT OxTOF could not be evaluated for ZnO NM-110; moreover, the physical–chemical properties, oxidation number, ionic potential, surface reducibility and redox reactivity reported in the literature are consistent with its high *in vitro* toxicity.^121^ Nevertheless, ZnO NM-110 is a complex ENM because its surface reactivity has biocidal properties,^122^ but its mode of action is essentially by dissolution.^121^

CuO-SA has the highest reactive site surface density in the series although not the most sites per gram and shows the highest OxTOF (Fig. 2D and 4E) along with CuFe_2_O_4_-SA. This correlates with their inflammatory effects, commonly associated with ROS generation and oxidative stress, making these ENMs the most toxic in the series.^123^ CuO-SA's oxidative damage was evaluated in HepG2 cells, with endocytosis transporting nanoparticles to endo/lysosomes, leading to lysosome disruption and copper ion overload.^124^ This mechanism may involve surface reactivity, which is initially overlooked due to lack of information on reactive sites. CuO-SA induced oxidative changes in A549 cells, increasing protein carbonylation, oxidizing protein thiols, and decreasing cell viability, with no effects from dissolved copper ions.^125^ These effects were more pronounced in CuO-SA with higher crystalline defects and ROS production likely due to higher reactive site surface density.^125^ Other studies reported CuO-SA's distinct cytotoxicity in A549 and HeLa S3 cells from direct interactions with cellular components, facilitated by greater surface area and reactive site density compared to microparticles. This parameter could facilitate the surface interactions of CuO-SA with its surroundings. Therefore, elevated intracellular levels can disrupt copper homeostasis, leading to pro-oxidative reactions^126^ produced at the surface of CuO-SA nanoparticles.^127^

Co_3_O_4_-SA nanoparticles exhibited higher reactivity for DTT depletion than the bulk material, which was reflected in the genotoxic effects of V79 cells and was primarily mediated by reactive oxygen species. This indicates that nano-sized Co_3_O_4_-SA induces significant cytotoxicity and DNA damage, unlike larger particles.^101^ The toxicity of TiO_2_ NM-101 is not fully understood due to inconsistent evidence across different tests.

In the case of CeO_2_, the literature has typically linked its toxicity with reactive oxygen species generation and the Ce^3+^/Ce^4+^ ratio,^128^ related to the exposed phase of ceria^129,130^ and its defects, which are also key for catalytic activity.^108^ These properties can be easily determined by several techniques.^131–135^ CeO_2_ NM-211, with redox surface sites and moderate oxidative capacity, causes cell death by apoptosis and DNA damage in pulmonary cell lines.^136,137^ The OxTOF of CeO_2_ NM-211 is between those of the titania ENMs and CuO-SA, despite its low BET area. This is consistent with the intense formaldehyde production in MeOH-TPSR, maximum at 259 °C, and with the characterization reported in the literature: CeO_2_ NM-211 surface contains 22% Ce(iii) (XPS), indicating redox sites, which induce ROS generation, as detected by ESR.^131–135^ CeO_2_ is highly oxidizing, while defect-rich CeO_2−*x*_ has antioxidant properties. This versatility is used to engineer ceria nanoparticles by tuning their properties^138,139^ and, thus, their performance (*e.g.* in catalysis), from combustion to selective oxidation,^131,140,141^ and in biomedical applications, from biocidal to antioxidant.^138,139,142^ The dynamic states of ceria nanomaterials in aqueous media^143^ or biological media^132,144,145^ resulting in defective ceria have been extensively investigated.^146^

ZnO NM-110, which induces protein carbonylation,^147^ has a high reactive site surface density, facilitating the formation of a protein corona. This aligns with reports on BSA–ZnO interactions, which demonstrate that protein adsorption on the ZnO NM-110 surface is higher compared to other ENMs, such as TiO_2_ NM-110.^84^ Despite TiO_2_ NM-110 having a larger surface area, its reactive site surface density is lower than that of ZnO NM-110.

Titania and iron oxide exhibit the lowest reactivity. Fe_3_O_4_-SA showed no adverse effects even after being internalized within the cells following 12 h exposure. Similarly, both titania samples convert methanol into carbon dioxide, but above 400 °C and to a limited extent, because they are essentially acidic. This low redox reactivity is consistent with their low DTT OxTOF. In line with our hypothesis, the high BET area of TiO_2_ NM-101 does not directly correlate with adverse effects. Although it has a high surface area (a physical feature), its chemical reactive profile counterpart does not run in parallel. The number of surface reactive sites is low, and their reactivity is moderate. TiO_2_ NM-101 is a relatively safe ENM, with no cytotoxicity for cell viability in immune, hepatic, reproductive and pulmonary cell lines, such as A549, HepG2, HK-2 or C3A. There are no toxicological data for TiO_2_-DT51, but the lower number and reactivity of its sites predict that DT51 would be safer than NM-101. Fe_3_O_4_, classified as low redox reactive *via* DTT OxTOF, is described as a safe ENM in terms of *in vitro* evaluation in the literature.^99^

An *in chemico* classification of ENMs can thus be proposed based on methanol chemisorption, reactivity of surface sites and DTT oxidative turnover frequency that may correlate with *in vitro* toxicity site-based dose metrics: CuO-SA ≈ CuFe_2_O_4_-SA > Co_3_O_4_-SA ≈ ZnO NM-110 ≫ CeO_2_ NM-211 ≥ Fe_3_O_4_-SA ≈ TiO_2_ NM-110.

### 4.3 Reactive surface site-based dose metrics

Recently, some studies emphasized the critical importance of adopting dose metrics that reflect the relevance of surface and particle number when assessing the nanotoxicity of ENMs, as traditional mass-based dose metrics are insufficient for evaluating the unique toxicological responses of nanoscale particles.^148,149^ These studies collectively underscore the need for more accurate dose metrics to assess the potential risks associated with ENMs. Due to the assumption that not all physical areas are equally populated by equally reactive sites, the reactive site concentration is proposed as a tool to better quantify the ENM exposition. For example, TiO_2_ NM-101 has a 4 times higher specific number of reactive sites than CeO_2_ NM-211 (2.8 *vs.* 0.7 mmol g^−1^) but also a 3 times higher surface area, so the reactive sites surface density is only slightly higher for the titania (7 *vs.* 6 sites per nm^2^), and therefore the comparison is similar to that of DTT OxTOF and OP_area_: TiO_2_ NM-101 with 3–4 times less oxidative potential than CeO_2_ NM-211. A different impression is provided by OP_mass_. Hence, because mass or physical surface area does not provide site-relevant dose metrics, the number of reactive sites connects with reactivity-triggered effects. This may serve as a new possible dose metric for assessing exposure to nanomaterials. The differences are greater when TiO_2_ NM-101 is compared to CuO-SA, with a very low specific surface area, and thus high reactive site surface density. These are much more reactive than those of titania and ceria, as observed by DTT OxTOF, but not as much as OP_area_ indicates. Moreover, dose metrics based on reactive sites underscore that CuO-SA, with a dose of sites of 4 μmol L^−1^, can produce a significant adverse effect in A549, while 280 μmol L^−1^ of titania sites did not significantly decrease cell viability.

## 5 Conclusions and outlook

Reactive-based nanotoxicity is primarily governed by the surface chemistry of engineered nanomaterials, making catalysis science principles highly relevant to describing the reactive nature of ENMs. Our study's findings are as follows. 1) The specific surface area does not reliably correlate with nanomaterial reactivity, necessitating consideration of surface site quantity, nature, and reactivity for categorization and site-specific dosing. 2) To address this reactive categorization and the specific-dosing approach, a new approach methodology that quantifies and describes reactive surface sites by chemisorption and reaction tests using probe molecules. 3) Methanol offers triple benefits, namely, it quantifies surface sites through chemisorption, characterizes surface reactivity (acidic, basic, or redox) *via* temperature-programmed surface reaction, and overcomes limitations of liquid-phase reactions, such as possible ion release, pH-dependent agglomeration, effects of the dispersion protocol, or stability issues, providing insights into the primary reactivity of thermally stable nanomaterials, such as metal oxides. 4) Combining site quantification with physiologically relevant oxidation reactions, such as DTT, allows for calculating site-specific oxidative reactivity (OxTOF), aiding nanomaterial classification. CuO-SA, CuFe_2_O_4_-SA, and Co_3_O_4_-SA are the most oxidizing ENMs according to a higher *in vitro* toxicity, while less reactive ENMs do not produce adverse effects in *in vitro* models.

CuO-SA, CuFe_2_O_4_-SA, Co_3_O_4_-SA, Fe_3_O_4_-SA, ZnO NM-110, CeO_2_ NM-211 and two TiO_2_ ENMs (DT51 and NM-101) are ranked into three categories with statistically different reactivity based on DTT. This fundamental site-specific reactivity information is a relevant descriptor for grouping ENMs and, ultimately, for understanding nanotoxicity. Moreover, the behavior of a given material not only depends on its specific nanoform (*e.g.*, crystallinity, size, band gap, solubility, hydrophobicity, surface charge, aspect ratio or shape) but also on its chemical reactive features, such as the number of surface reactive sites, their nature, their reactivity and their relative populations. In other cases, the adverse effect is not related to the reactivity but to other features, such as in multiwalled carbon nanotubes.^150^

This new methodology offers a complementary *in chemico* approach to unravel nanomaterial modes of action. To validate its effectiveness, further testing with additional reference and real-life ENMs as well as relevant and comparable toxicity information are essential. On a broader vista, the correlation with cellular assays will help establish molecular insight on the reactive basis of nanotoxicity. There are, however, significant structure, reactivity and toxicity data gaps to connect adverse effects with chemical reactivity. This approach aims to elucidate the specific pathways impacted by ENMs, highlighting their role in achieving a comprehensive understanding of nanomaterial toxicity and advocating safe-by-design principles. Filling these data gaps is part of the mission of nanoinformatics and nanosafety projects supported by platforms such as eNanoMapper. Mapping all reactive properties enables a more relevant grouping of nanomaterials because the acidic, basic and redox properties not only reflect their reactivity for adverse effects but also for interaction with species and molecules in biological systems.

## Data availability

All data supporting the findings of this study are presented in the main article and the ESI.†

## Author contributions

M. A. Bañares: conceptualization, original idea, supervision, writing – original draft preparation; V. Alcolea-Rodriguez: data curation, writing – original draft preparation, investigation, statistical analysis, experimental methodology; R. Portela: analysis, planning, supervision, writing – original draft preparation. V. Calvino-Casilda: reviewing and editing. All authors equally contributed to the writing.

## Conflicts of interest

All authors have given approval to the final version of the manuscript. The authors declare no competing financial interest.

## Supplementary Material

EN-011-D3EN00810J-s001

EN-011-D3EN00810J-s002
