# Supplementary material for: In chemico methodology for engineered nanomaterial categorization according to number, nature and oxidative potential of reactive surface sites
Source: Environ Sci Nano. 2024 Jul 9;11(9):3744–60. doi: 10.1039/d3en00810j (PMC11392058; doi:10.1039/d3en00810j)
Supplement: EN-011-D3EN00810J-s001 [file EN-011-D3EN00810J-s001.pdf]

Electronic Supplementary Material (ESI) for Environmental Science: Nano.
This journal is © The Royal Society of Chemistry 2024

In chemico methodology for engineered nanomaterials categorization according to number, nature and oxidative capacity of reactive surface sites

V. Alcolea-Rodriguez[a*], R. Portela[a], V. Calvino-Casilda[b], M.A. Bañares[a*]

[a] Instituto de Catálisis y Petroleoquímica, ICP-CSIC, Marie Curie 2, 28049-Madrid, Spain

[b] Departamento de Ingeniería Eléctrica, Electrónica, Control, Telemática y Química Aplicada a la Ingeniería, E.T.S. de Ingenieros Industriales, UNED, Juan del Rosal 12, 28040-Madrid, Spain

***Corresponding authors**: miguel.banares@csic.es victor.alcolea@csic.es

SUPPORTING INFORMATION

1 Reactive sites calculation from methanol chemisorption 2

2 Methanol-TPSR (Temperature-Programmed Surface Reaction) reaction equations .. 2

3 Figures 4

*Figure S 1. Probe reactions for quantitative reactive characterization of NMs: **A**) Methanol chemisorption on the surface of a NMs with formation of a methoxy group per active site, followed by surface reaction and products desorption; redox sites lead to formaldehyde formation, basic sites produce carbon dioxide, and two nearby acid sites generate dimethyl ether. **B)** Oxidation of dithiothreitol (DTT) catalysed by a nanoparticle (NP) and quantification of non-oxidized DTT with Ellman's reagent (DTNB) by UV-Vis spectrophotometry detection at 412 nm of the coloured product.*

Figure S 2 Experimental setup for methanol chemisorption and temperature-programmed surface reaction (TPSR)

Figure S 3. Methanol temperature-programmed surface reaction (TPSR) for SiC (blank)

Figure S 4 Calibration for DTNB-DTT complex measured to 412 nm

Figure S 5 Methanol-TPSR reactive profile for bulk Co_3O_4 microparticles

Figure S 6 DTT conversion and NIOG values for the NMs evaluated

4 Tables 6

***Table S 1** Physicochemical properties reported in the literature for TiO_2 DT51, TiO_2 NM-101, CeO_2 NM-211, ZnO NM-110 and CuO from Sigma Aldrich.*

***Table S 2.** In vitro toxicity data reported in the literature for TiO_2 NM-101, CeO_2 NM-211, ZnO NM-110 and CuO indicating for a given exposure time the concentration at which a NM induces effects significantly different to control ($p<0.05$, 0.01 or 0.001) or the half-maximal effective concentration (EC_{50})*

***Table S3**. Data of methanol chemisorption*

5 Bibliography 10

1 Methanol chemisorption and reactive sites calculation

The number of surface reactive sites equals the total number of methanol molecules that are absorbed upon saturation that are not release unreacted.

The clean dehydrated sample under inert argon results in no methanol signal. The m/z = 31 signal of methanol must be checked under inert flow (zero) and in by-pass at the constant concentration and flow rate used for chemisorption (2000 ppm methanol, 100 NmL/min flow rate, controlled by a mass flow controller) before starting the experiment to have a linear calibration. When 2000 ppm CH_3OH in 5%He/Ar is fed, we quickly observe the sharp rise of helium signal, indicating time zero of chemisorption; then we observe the rise of H_2O m/z 18 signal, indicative of the chemisorption of methanol. No methanol flows out at this moment in time for it is chemisorbing. As the surface reaches its maximum capacity of methanol chemisorption, there is a decrease in the online H_2O signal, and once the surface is saturated with methanol, new methanol molecules that enter the system do not have a binding site and thus leave the reactor, as observed by online methanol m/z 31 signal increase. When the sample is saturated with methoxy, the feed is switched to 100 mL/min Ar to first purge residual methanol vapor, and then run the TPSR of the chemisorbed methoxy species by linearly heating the sample from 100 to 450 ºC at 10 ºC/min. Thise signal must be integrated during the chemisorption step to quantify the moles or molecules of methanol retained by the material on the surface until saturation ($MeOH_s$), and during the TPSR step to calculate the fraction of methanol that is released unreacted ($MeOH_u$), as follows:

When methanol is fed to the reactor (t=0) for chemisorption the signal in the reactor outlet is initially low (or even 0), because methanol is mainly (or even totally) adsorbed on the nanomaterial. Outlet concentration of methanol progressively increases as the surface sites are filled. When the surface is saturated (t=s), methanol signal remains constant and equal to the by-pass value, corresponding to 2000 ppm. $MeOH_s$ is determined by calculating the total amount of methanol fed to the reactor from t=0 to t=s and subtracting the integrated signal of methanol in the same period, corresponding to the released methanol that was not adsorbed. Methanol molar flow is readily calculated from its concentration and controlled flow rate (100 mL/min). Upon heating, the fraction of methanol that is released unreacted is calculated by integrating the methanol signal. The reacted methanol ($MeOH_r$) is then obtained by subtracting both values, and is equal to the number of surface sites. The specific number of active sites is calculated by dividing this number by the mass of NM used for the experiment. The specific number of surface sites divided by the NM BET area delivers the active sites surface density.

2 Methanol-TPSR (Temperature-Programmed Surface Reaction) reaction equations

The sample saturated with methoxy and purged in Ar is linearly heated from 100 to 450 ºC at 10 ºC/min for TPSR. The m/z values followed in the residual gas analyzer were: CH_3OH (methanol) = 31, HCHO (formaldehyde) = 30, CH_3OCH_3 (dimethyl ether, DME) = 45, CH_3OOCH (methylformate) = 60, $(CH_3O)_2CH_2$ (dimethoxy methane) = 75, H_2O (water) = 18, and CO_2 (carbon dioxide) = 44. Methylformate, dimethyl ether and formaldehyde signals were corrected for the contributions of methanol and carbon monoxide to the selected m/z values.

The *type* and *amount* of products and the *temperature* at which they are detected correlate with the *nature*, *amount* and *reactivity* of the surface sites, respectively (Figure S 1A): oxidation of methoxy species to formaldehyde (HCHO) is indicative of redox sites (Eq. S1); dehydration of two adjacent methoxy to dimethyl ether (CH_3OCH_3), of acidic sites (Eq. S2); and formation of carbon dioxide (CO_2), of basic sites, which strongly bind methanol that is typically burnt and desorbed at high temperatures (Eq. S3); highly reactive redox sites will also produce CO_2 due to fast formaldehyde combustion (over-oxidation reaction), but at significantly lower temperature.[1] Bifunctional sites may be present at the surface of NMs if two kind of reactive sites are in close vicinity, generating different products: methyl formate ($HCOOCH_3$) originates from basic-redox sites (Eq. S4) and dimethoxymethane ($(CH_3O)_2CH_2$) from acid-redox sites (Eq. S5). Water and CO are secondary products formed in several reactions and may not be correlated with a specific site. On occasions, less reactive sites release unreacted methoxy species as methanol, which typically occurs at low temperatures in the TPSR profile.

<u>- Single sites:</u>

- Redox sites: $CH_3OH + \frac{1}{2}O_2 \rightarrow HCHO + H_2O$ (Eq. S1)
- Acid sites: $2CH_3OH \leftrightarrow CH_3OCH_3 + H_2O$ (Eq. S2)
- Basic or redox (over-oxidation) sites: $CH_3OH + \frac{3}{2}O_2 \rightarrow CO_2 + 2\,H_2O$ (Eq. S3)

<u>- Bifunctional sites:</u>

- Basic-redox sites: $2CH_3OH + O_2 \leftrightarrow HCOOCH_3 + 2\,H_2O$ (Eq. S4)
- Acid-redox sites: $3CH_3OH + \frac{1}{2}O_2 \rightarrow CH_2(OCH_3)_2 + 2H_2O$ (Eq. S5)

3 Figures

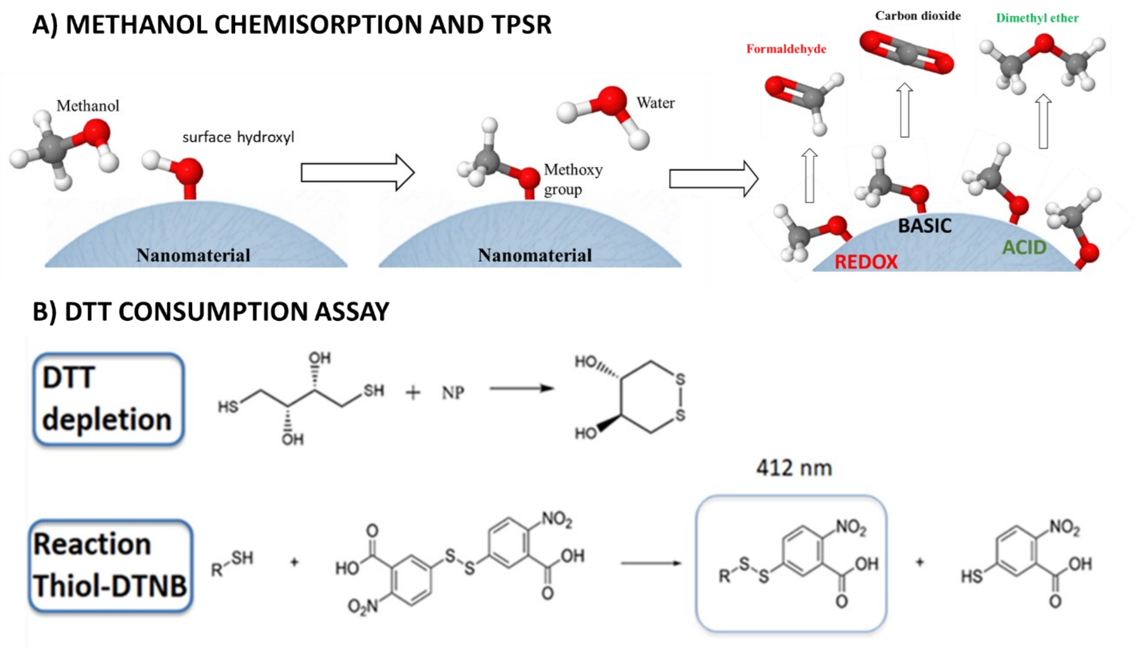

*Figure S1. Probe reactions for quantitative reactive characterization of NMs**:** **A**) Methanol chemisorption on the surface of a NMs with formation of a methoxy group per active site, followed by surface reaction and products desorption; redox sites lead to formaldehyde formation, basic sites produce carbon dioxide, and two nearby acid sites generate dimethyl ether. **B)** Oxidation of dithiothreitol (DTT) catalysed by a nanoparticle (NP) and quantification of non-oxidized DTT with Ellman's reagent (DTNB) by UV-Vis spectrophotometry detection at 412 nm of the coloured product.*

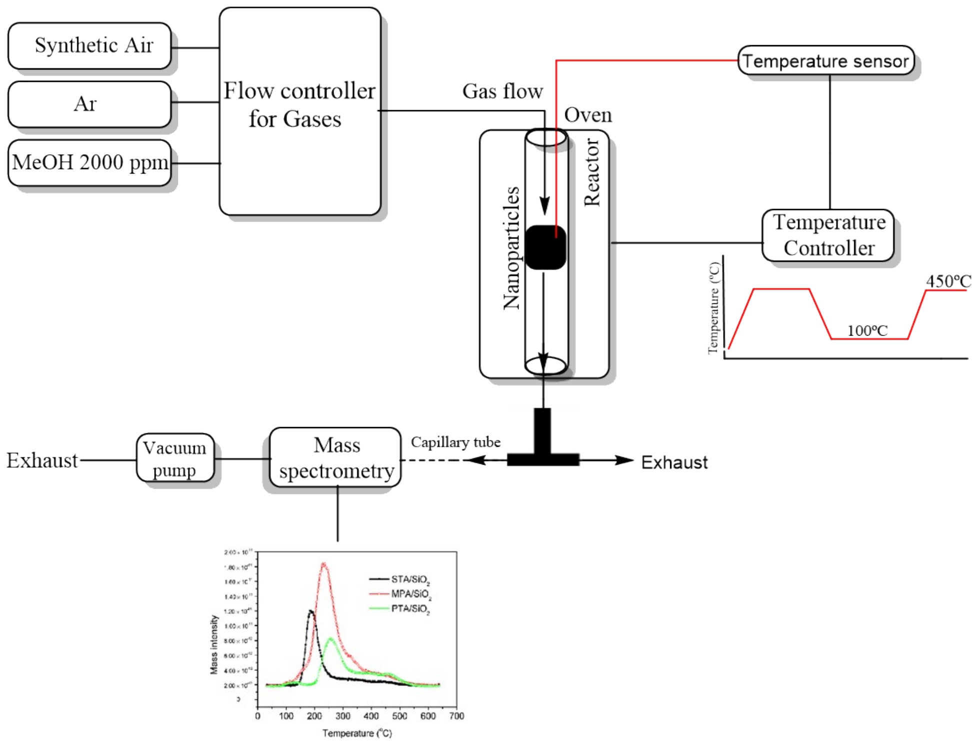

Figure S2. Experimental setup for methanol chemisorption and temperature-programmed surface reaction (TPSR)

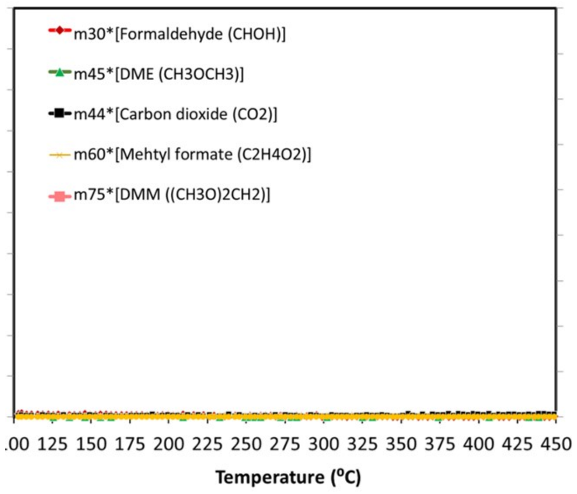

Figure S3. Methanol temperature-programmed surface reaction (TPSR) for SiC (blank)

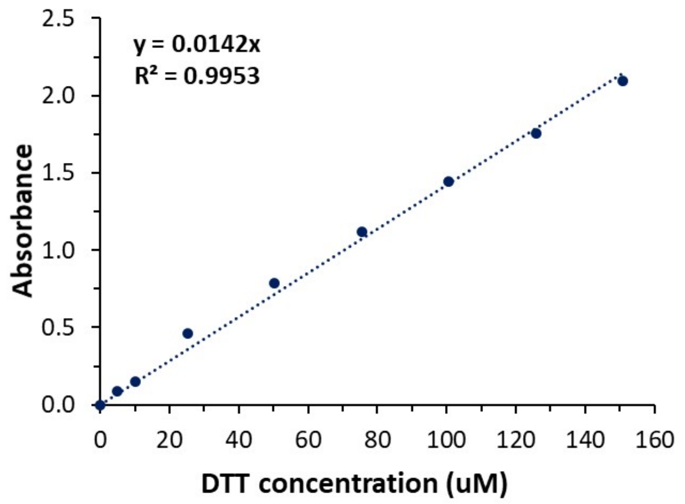

Figure S4. Calibration for DTNB-DTT complex measured to 412 nm

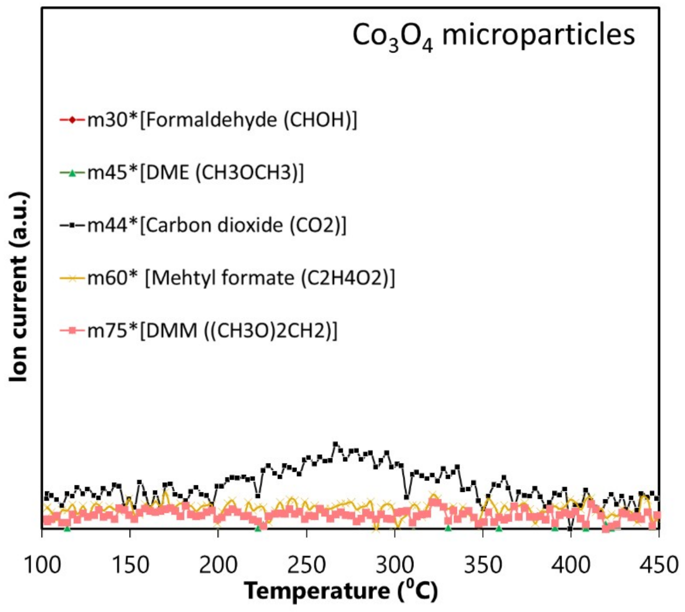

Figure S5. Methanol-TPSR reactive profile for bulk Co_3O_4 microparticles

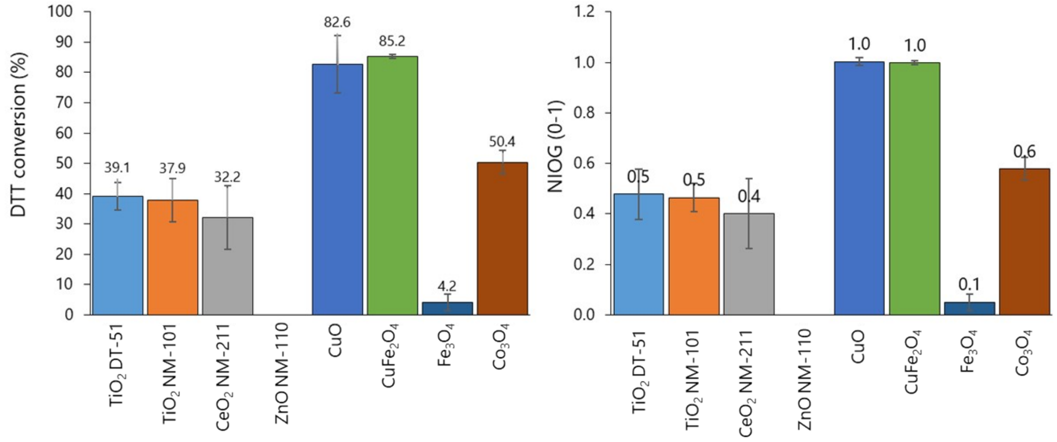

Figure S6. DTT conversion and NIOG values for the NMs evaluated

4 Tables

***Table S1** Physicochemical properties reported in the literature for TiO_2 DT51, TiO_2 NM-101, CeO_2 NM-211, ZnO NM-110 and CuO from Sigma Aldrich.*

Powder NM	Supplier code	Particle size (nm)	Particle size distribution (nm)	Specific surface area (m^2/g)	Shape	Other information	Ref.
**TiO_2 DT51**	CristalAC TiV™ DT-51	NA	NA	90	-	Anatase,<br>SO_3: 1.25 wt%<br>pH = 3	2
**TiO_2 NM-101**	JRCNM01001a	5-6	<100 → 95%<br><50 → 77 %<br><10 → 11 %	170/316	Equiaxed and rounded -or slightly elongated- primary particles	Anatase<br>Thermal synthesis<br>photocatalytic activity	3,4
		9	4-8 to 50-100	322			5
		8	NA	320			6
**CeO_2 NM-211**	JRCNM02101a	<10 to 20	In water:<br>D_{10} 810 ± 160<br>D_{50} 202 ± 17<br>D_{90} 130 ± 60	65	Homogeneous near spherical primary particles	Precipitated	3,7
		10.3	NA	66		Ce:O = 1:2 (XPS)	8
**ZnO NM-110**	JRCNM62101a	70-90	NA	12.4	Primary particles: bottle-like, rod-shaped, rectangular particles (215 nm x 66 nm and 115 nm x 40 nm) and sticks (180 nm x 23 nm)	ζ: -19.1 ± 0.5 mV<br>Zincite	3,9
		202.6 ± 12.8	NA	14			10,11
**CuO**	SigmaAldrich: 544868	<50	NA	NA	Spherical and smooth surface	Complexometric EDTA:<br>77-82.6 % Cu	12
		NA	NA	11		Tenorite	13,14
		37	NA	11		Crystallite size: 17 nm<br>IEP: 8.3	15
		33.3 ± 10.7	10-100 nm	NA		ζ: 15.1 ± 9.4 mV	16
		55	20-80 nm	NA			17
**$CuFe_2O_4$**	SigmaAldrich: 641723	35	In water: 810<br>In CCM: 387	NA	Spherical	ζ in water: 16 mV	18
**Co_3O_4**	SigmaAldrich: 637025	11.5-56.4	In water:<br>207+7.8 (20 µg/mL)	30.5	Prevalently elongated-hexagonal	ζ in water: -23.9 mV	19
**Fe_3O_4**	SigmaAldrich: 637106	33 ± 14.58	In CCM: 858.5	NA	Spherical	ζ in CCM: -11 mV	20

***Table S2.** In vitro toxicity data reported in the literature for TiO_2 NM-101, CeO_2 NM-211, ZnO NM-110 and CuO indicating for a given exposure time the concentration at which a NM induces effects significantly different to control ($p<0.05$, 0.01 or 0.001) or the half-maximal effective concentration (EC_{50})*

NM	Cell line[1]	Test[2]	Parameter	Time (h)	Concentration (µg/mL)	Ref.
**CuO #544848 Sigma Aldrich (CuO-SA)**	A549	MTT	p<0.001	3	10	21
		MTT	p<0.001	24	5	
		NRU	p<0.001	3	50	
		NRU	p<0.001	24	10	
		IL-8 release	p<0.001	3	100	
		IL-8 release	p<0.001	24	10	
		MTT	EC_{50}	24	17.75	
		CFA	p<0.001	24	10	22
	HeLa S3	CFA	p<0.001	24	10	
	A549	MTT	p<0.05	6	100	14
		MTT	p<0.05	24	10	

		MTT	p<0.05	1	25	16
		MTT	p<0.05	3	25	
**ZnO NM-110 (JRCNM62101a)**	RAW 264.7	Resazurin	EC_{50}	24 - 72	12.16-14.72	23
		NRU	EC_{50}	24 - 72	18.40-25.48	
	MH-S	Resazurin	EC_{50}	24 - 72	22.08-17.92	
		NRU	EC_{50}	24 - 72	19.68-23.04	
	TM3	WST-1	EC_{50}	24	10.06	
	TM4	WST-1	EC_{50}	24	11.88	
	16HBE	WST-8	EC_{50}	24	17.79	
	HUVEC	WST-1	p<0.001	24	32	11
		NRU	p<0.001	24	32	
	A549	Resazurin	EC_{50}	24	76	24,25
		Resazurin	p<0.05	24	48	
		Resazurin	EC_{50}	3	215	
		Resazurin	p<0.05	3	120	
	TK6	Comet assay, DNA damage	Saturation, high damage	3	14	25
		Comet assay, DNA damage	Saturation, high damage	24	14	
	A549	Comet assay, DNA damage	p<0.0001	3	14	
	Caco-2	MTT	EC_{50}	24	25	26
		WST-1	EC_{50}	24	28	
	PMA treated THP-1	WST-1	EC_{50}	24	8.5	27
		LDH	EC_{50}	24	6.4	
		Resazurin	EC_{50}	24	9.5	
	C3A	WST-1	EC_{50}	24	5-10 $\mu g/cm^2$	5
		Resazurin	EC_{50}	24	ca.10 $\mu g/cm^2$	
		IL-8	p<0.05	24	0.31 $\mu g/cm^2$	
**TiO_2 NM-101 (JRCNM1001a)**	A549	Alamar blue	p<0.05	24	Not reached up to 100	28
		WST-1	EC_{50}	24	Not reached up to 80 $\mu g/cm^2$	29
	HepG2	WST-1	EC_{50}	24		
	HK-2	WST-1	EC_{50}	24		
	C3A	WST-1	EC_{50}	24	Not reached up to 80 $\mu g/cm^2$	5
		IL-8	p<0.05	24	20 $\mu g/cm^2$	
	HUVEC	IL-6	p<0.05	24	Not reached up to 32	11
		TNF-α	p<0.05	24		
		WST-1	p<0.05	24		
		NRU	p<0.05	24		
	BEAS-2B	Trypan blue	p<0.05	24	Not reached up to 100	30
		Fpg-Comet assay	p<0.05	24	10 Direct damage	
		Fpg-Comet assay	p<0.05	24	100 Oxidative damage	
		Micronucleous assay	p<0.001	24	Not reached up to 100	
		IL-6	p<0.05	24	100	
		IL-8	p<0.05	24	Not reached up to 100	
		TNF-α	p<0.05	24		
	RAW 264.7 macrophages	MTS	p<0.05	24		
		IL-6	p<0.05	18	10	
		TNF-α	p<0.05	18	100	
**CeO2 NM-211 (JRCNM02101a)**	NR8383	LDH	p<0.05	16	90	31
		GLU	p<0.05	16	180	
		TNF-α	p<0.05	16	22.5	
		Extracellular H_2O_2/ROS	p<0.05	1.5	Not reached up to 180	
	H4IIE	MTT	p<0.05	24	6.25	32
		MTT	p<0.05	72	0.098	
		NRU	p<0.05	24	Not reached up to 100	

		NRU	p<0.05	72	Not reached up to 100	
		LDH	p<0.05	24	Not reached up to 100	
		LDH	p<0.05	72	100	
	RTG-2	NRU and MTT	p<0.05	24	Not reached up to 100	
		NRU and MTT	p<0.05	72		
**$CuFe_2O_4$ #641723 SigmaAldrich ($CuFe_2O_4$-SA)**	A549	NRU and MTT	p<0.05	24	10	18
	HepG2	NRU and MTT	p<0.05	24	10	
	A549	Intracellular ROS (DCFH-DA)	p<0.05	24	50	
	HepG2	Intracellular ROS (DCFH-DA)	p<0.05	24	50	
	A549	Intracellular GSH	p<0.05	24	50	
	HepG2	Intracellular GSH	p<0.05	24	50	
	A549	MMP	p<0.05	24	50	
	HepG2	MMP	p<0.05	24	50	
**Co_3O_4 #637025 SigmaAldrich (Co_3O_4-SA)**	A549	TrypanBlue	p<0.05	24	Not reached up to 40	19
	A549	WST-1	p<0.05	24	40	
	BEAS-2B	TrypanBlue	p<0.05	24	40	
	BEAS-2B	WST-1	p<0.05	24	5	
	A549	LDH	p<0.05	24	Not reached up to 40	
	BEAS-2B	LDH	p<0.05	24	40	
	A549	Fpg-Comet DNA damage	p<0.05	24	20	
	BEAS-2B	Fpg-Comet DNA damage	p<0.05	24	5	
	A549	IL-6	p<0.05	24	Not reached up to 40	
	A549	IL-8	p<0.05	24	Not reached up to 40	
	A549	TNF-alpha	p<0.05	24	Not reached up to 40	
	BEAS-2B	IL-6	p<0.05	24	Not reached up to 40	
	BEAS-2B	IL-8	p<0.05	24	20	
	BEAS-2B	TNF-alpha	p<0.05	24	Not reached up to 40	
**Fe_3O_4 #637106 SigmaAldrich (Fe_3O_4-SA)**	A549	MTT	p<0.05	24	Not reached up to 100	20
	A549	MTT	p<0.05	48	Not reached up to 100	
	A549	MTT	p<0.05	72	Not reached up to 100	
	A549	Internalization (TEM)	-	12	Positive at 50	

[1]Cell lines: *A549 (adenocarcinomic human alveolar basal epithelial cells), HeLa S3 (Human cervix carcinoma cell line), Raw 264.7 (semi-adherent macrophage-like cell line derived from BALB/c mice), MH-S(murine alveolar macrophages), TM3 and TM4 (proliferating mouse Leydig cell line), 16HBE (human bronchial epithelial cell line), HUVEC (Human Umbilical Vein Endothelial Cells), TK6 (Human lymphoblastoid, In vitro mammalian cells), Caco-2 (human colorectal adenocarcinoma cells), PMA treated THP-1 (human leukemia monocytic cell line differentiated into macrophage-like), C3A (human liver adherent cells), HepG2 (human liver cancer cell line), BEAS-B2 (immortalized but non-tumorigenic epithelial cell line from human bronchial epithelium), NR8383 (alveolar macrophages), H4IIE (rat hepatoma cell line), RTG-2a (fibroblast morphology cell line from rainbow trout ovary).*
[2]Tests: *MTT = (3-(4,5-dimethylthiazol-2-yl)-2,5-diphenyltetrazolium bromide, IL-8 = Interleukin 8 release, CFA = Colony Forming Ability, NRU = neutral red uptake, LDH = Lactate Dehydrogenase, GLU = β-glucuronidase, TNF-α = Tumor Necrosis Factor, MMP = Mitochondrial membrane potential assay*

Table S3. *Data of methanol chemisorption*

Nanomaterial	Time until surface saturation (min)	Ratio methanol introduced/methanol chemisorbed (%)
TiO_2 DT51	59.22	43
TiO_2 NM-101	70.21	48
CeO_2 NM-211	68.19	32
ZnO NM-110	46.57	11
CuO	42.83	26
$CuFe_2O_4$	36.23	34
Co_3O_4	34.51	23
Fe_3O_4	50.28	23

5 Bibliography

1 Jehng JM, Wachs IE, Patience GS, Dai YM. Experimental methods in chemical engineering: Temperature programmed surface reaction spectroscopy—TPSR. *Canadian Journal of Chemical Engineering* 2021; **99**: 423–434.

2 ACTIV C. CristalACTiV™ TiO2 DT-51 - Product data sheet. 2012.

3 JRC NANOMATERIALS REPOSITORY. List of Representative Nanomaterials. 2016.

4 Rasmussen K, Mast J, Temmerman P De, Verleysen E, Waegeneers N, Steen F Van *et al. Titanium Dioxide, NM-100, NM-101, NM-102, NM-103, NM-104, NM-105: Characterisation and Physico- Chemical Properties*. 2014 doi:10.2788/79554.

5 Kermanizadeh A, Pojana G, Gaiser BK, Birkedal R, Bilaničová D, Wallin H *et al.* In vitro assessment of engineered nanomaterials using a hepatocyte cell line: Cytotoxicity, pro-inflammatory cytokines and functional markers. *Nanotoxicology* 2013; **7**: 301–313.

6 Sauer UG, Vogel S, Aumann A, Hess A, Kolle SN, Ma-Hock L *et al.* Applicability of rat precision-cut lung slices in evaluating nanomaterial cytotoxicity, apoptosis, oxidative stress, and inflammation. *Toxicol Appl Pharmacol* 2014; **276**: 1–20.

7 Singh C, Europäische Kommission Gemeinsame Forschungsstelle Institute for Health and Consumer Protection. *Cerium Dioxide NM-211, NM-212, NM-213, characterisation and test item preparation JRC repository: NM-series of representative manufactured nanomaterials*. 2014 doi:10.2788/80203.

8 Sauer UG, Vogel S, Aumann A, Hess A, Kolle SN, Ma-Hock L *et al.* Applicability of rat precision-cut lung slices in evaluating nanomaterial cytotoxicity, apoptosis, oxidative stress, and inflammation. *Toxicol Appl Pharmacol* 2014; **276**: 1–20.

9 Singh C, Friedrichs S, Levin M, Birkedal R, Jensen KA, Pojana G *et al. Zinc Oxide NM-110, NM-111, NM-112, NM-113 Characterisation and Test Item Preparation*. 2011 doi:10.2787/55008.

10 Kermanizadeh A, Vranic S, Boland S, Moreau K, Baeza-Squiban A, Gaiser BK *et al.* An in vitroassessment of panel of engineered nanomaterials using a human renal cell line: cytotoxicity, pro-inflammatory response, oxidative stress and genotoxicity. *BMC Nephrol* 2013; **14**: 96.

11 Gu Y, Cheng S, Chen G, Shen Y, Li X, Jiang Q *et al.* The effects of endoplasmic reticulum stress inducer thapsigargin on the toxicity of ZnO or TiO2 nanoparticles to human endothelial cells. *Toxicol Mech Methods* 2017; **27**: 191–200.

12 Life M. Copper oxide (ref. number: 544868) specifications. 2023.

13 Martín-Gómez J, Hidalgo-Carrillo J, Estévez RC, Urbano FJ, Marinas A. Hydrogen photoproduction on TiO2-CuO artificial olive leaves. *Appl Catal A Gen* 2021; **620**: 118178.

14 Perelshtein I, Lipovsky A, Perkas N, Gedanken A, Moschini E, Mantecca P. The influence of the crystalline nature of nano-metal oxides on their antibacterial and toxicity properties. *Nano Res* 2015; **8**: 695–707.

15 Martín-Gómez J, Hidalgo-Carrillo J, Montes V, Estévez-Toledano RC, Escamilla JC, Marinas A *et al.* EPR and CV studies cast further light on the origin of the enhanced hydrogen production through glycerol photoreforming on CuO:TiO2 physical mixtures. *J Environ Chem Eng* 2021; **9**: 105336.

16 Moschini E, Colombo G, Chirico G, Capitani G, Dalle-Donne I, Mantecca P. Biological mechanism of cell oxidative stress and death during short-term exposure to nano CuO. *Sci Rep* 2023; **13**: 2326.

17 Kwon J-T, Kim Y, Choi S, Yoon B, Kim H-S, Shim I *et al.* Pulmonary Toxicity and Proteomic Analysis in Bronchoalveolar Lavage Fluids and Lungs of Rats Exposed to Copper Oxide Nanoparticles. *Int J Mol Sci* 2022; **23**: 13265.

18 Ahmad J, Alhadlaq HA, Alshamsan A, Siddiqui MA, Saquib Q, Khan ST *et al.* Differential cytotoxicity of copper ferrite nanoparticles in different human cells. *Journal of Applied Toxicology* 2016; **36**: 1284–1293.

19 Cavallo D, Ciervo A, Fresegna AM, Maiello R, Tassone P, Buresti G *et al.* Investigation on cobalt-oxide nanoparticles cyto-genotoxicity and inflammatory response in two types of respiratory cells. *Journal of Applied Toxicology* 2015; **35**: 1102–1113.

20 Solorio-Rodríguez A, Escamilla-Rivera V, Uribe-Ramírez M, González-Pozos S, Hernández-Soto J, Rafael-Vázquez L *et al.* In vitro cytotoxicity study of superparamagnetic iron oxide and silica nanoparticles on pneumocyte organelles. *Toxicology in Vitro* 2021; **72**: 105071.

21 Moschini E, Gualtieri M, Colombo M, Fascio U, Camatini M, Mantecca P. The modality of cell-particle interactions drives the toxicity of nanosized CuO and TiO2 in human alveolar epithelial cells. *Toxicol Lett* 2013; **222**: 102–116.

22 Semisch A, Ohle J, Witt B, Hartwig A. Cytotoxicity and genotoxicity of nano - and microparticulate copper oxide: role of solubility and intracellular bioavailability. *Part Fibre Toxicol* 2014; **11**: 10.

23 Farcal L, Andón FT, Di Cristo L, Rotoli BM, Bussolati O, Bergamaschi E *et al.* Comprehensive in vitro toxicity testing of a panel of representative oxide nanomaterials: First steps towards an intelligent testing strategy. *PLoS One* 2015; **10**: 1–34.

24 Precupas A, Gheorghe D, Botea-Petcu A, Leonties AR, Sandu R, Popa VT *et al.* Thermodynamic Parameters at Bio-Nano Interface and Nanomaterial Toxicity: A Case Study on BSA Interaction with ZnO, SiO2, and TiO2. *Chem Res Toxicol* 2020; **33**: 2054–2071.

25 Yamani N El, Collins AR, Rundén-Pran E, Fjellsbø LM, Shaposhnikov S, Zienolddiny S *et al.* In vitro genotoxicity testing of four reference metal nanomaterials, titanium dioxide, zinc oxide, cerium oxide and silver: Towards reliable hazard assessment. *Mutagenesis* 2017; **32**: 117–126.

26 Cao Y, Roursgaard M, Kermanizadeh A, Loft S, Møller P. Synergistic effects of zinc oxide nanoparticles and fatty acids on toxicity to caco-2 cells. *Int J Toxicol* 2015; **34**: 67–76.

27 Safar R, Doumandji Z, Saidou T, Ferrari L, Nahle S, Rihn BH *et al.* Cytotoxicity and global transcriptional responses induced by zinc oxide nanoparticles NM 110 in PMA-differentiated THP-1 cells. *Toxicol Lett* 2019; **308**: 65–73.

28 Precupas A, Gheorghe D, Botea-Petcu A, Leonties AR, Sandu R, Popa VT *et al.* Thermodynamic Parameters at Bio-Nano Interface and Nanomaterial Toxicity: A Case Study on BSA Interaction with ZnO, SiO2, and TiO2. *Chem Res Toxicol* 2020; **33**: 2054–2071.

29 Thongkam W, Gerloff K, van Berlo D, Albrecht C, Schins RPF. Oxidant generation, DNA damage and cytotoxicity by a panel of engineered nanomaterials in three different human epithelial cell lines. *Mutagenesis* 2017; **32**: 105–115.

30 Zijno A, Cavallo D, Di Felice G, Ponti J, Barletta B, Butteroni C *et al.* Use of a common European approach for nanomaterials' testing to support regulation: a case study on titanium and silicon dioxide representative nanomaterials. *Journal of Applied Toxicology* 2020; **40**: 1511–1525.

31 Wiemann M, Vennemann A, Sauer UG, Wiench K, Ma-Hock L, Landsiedel R. An in vitro alveolar macrophage assay for predicting the short-term inhalation toxicity of nanomaterials. *J Nanobiotechnology* 2016; **14**: 16.

32 Rosenkranz P, Fernández-Cruz ML, Conde E, Ramírez-Fernández MB, Flores JC, Fernández M *et al.* Effects of cerium oxide nanoparticles to fish and mammalian cell lines: An assessment of cytotoxicity and methodology. *Toxicology in Vitro* 2012; **26**: 888–896.
