# Supplementary figures and images for: In chemico methodology for engineered nanomaterial categorization according to number, nature and oxidative potential of reactive surface sites
Source: Environ Sci Nano. 2024 Jul 9;11(9):3744–60. doi: 10.1039/d3en00810j (PMC11392058; doi:10.1039/d3en00810j)

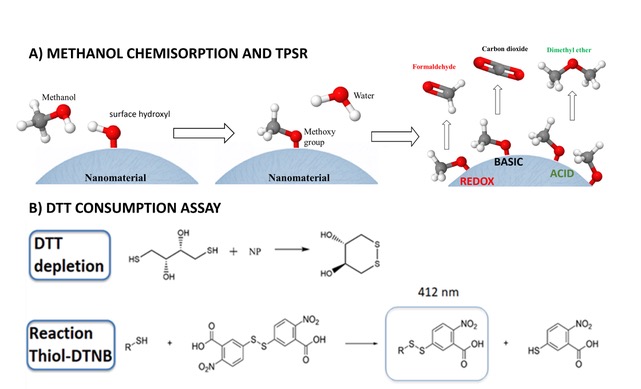

Supplement: EN-011-D3EN00810J-s002 [file EN-011-D3EN00810J-s002.zip › Figure S1.jpeg]

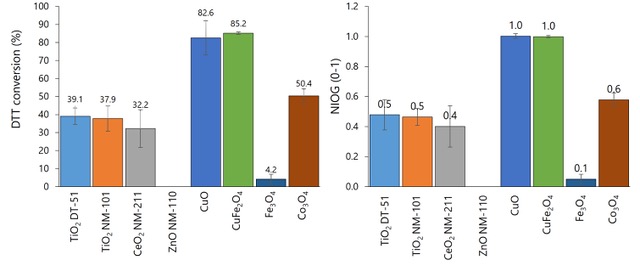

Supplement: EN-011-D3EN00810J-s002 [file EN-011-D3EN00810J-s002.zip › Figure S6.jpeg]

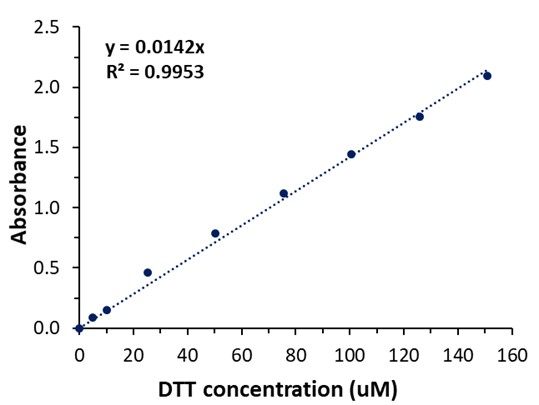

Supplement: EN-011-D3EN00810J-s002 [file EN-011-D3EN00810J-s002.zip › Figure S4.jpg]

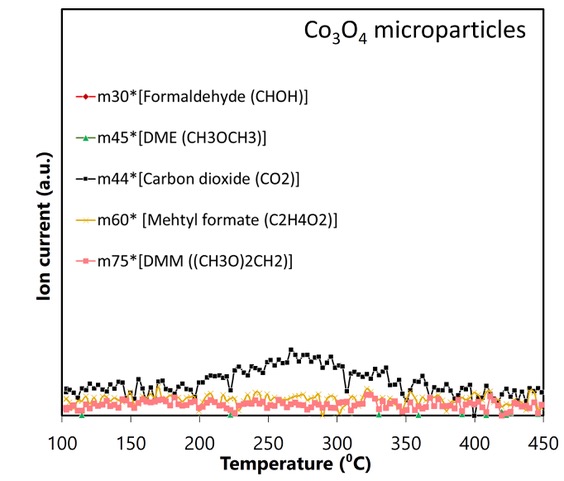

Supplement: EN-011-D3EN00810J-s002 [file EN-011-D3EN00810J-s002.zip › Figure S5.jpeg]

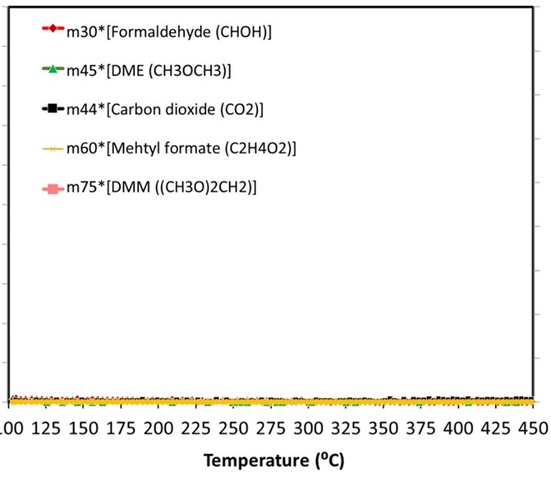

Supplement: EN-011-D3EN00810J-s002 [file EN-011-D3EN00810J-s002.zip › Figure S3.jpeg]

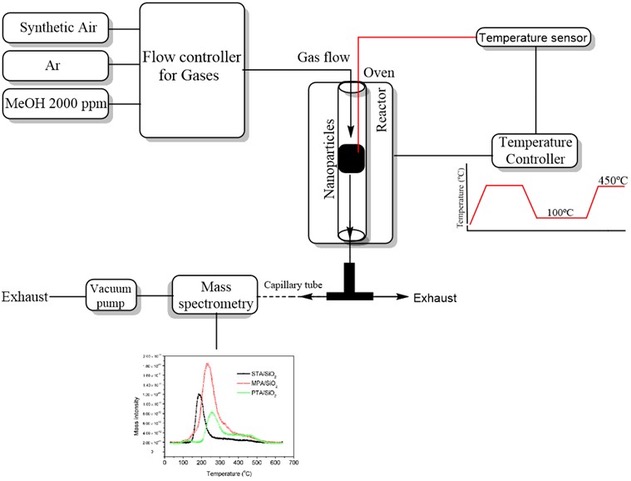

Supplement: EN-011-D3EN00810J-s002 [file EN-011-D3EN00810J-s002.zip › Figure S2.jpeg]
